# BTB/POZ domain‐containing protein 7/hypoxia‐inducible factor 1 alpha signalling axis modulates hepatocellular carcinoma metastasis

**DOI:** 10.1002/ctm2.556

**Published:** 2021-10-12

**Authors:** Kuan Hu, Juanni Li, Zhiming Wang, Yuanliang Yan, Yuan Cai, Bi Peng, Jinzhou Huang, Dongren He, Lei Zhou, Zhijie Xu, Yiming Tao

**Affiliations:** ^1^ Department of General Surgery Xiangya Hospital, Central South University Changsha Hunan China; ^2^ Department of Pathology Xiangya Hospital, Central South University Changsha Hunan China; ^3^ Department of Pharmacy National Clinical Research Center for Geriatric Disorders Xiangya Hospital, Central South University Changsha Hunan China; ^4^ Department of Oncology Mayo Clinic Rochester Minnesota USA; ^5^ Department of Anesthesiology Third Xiangya Hospital of Central South University Changsha Hunan China

Dear Editor,

This study is the first to demonstrate that HIF‐1α could activate BTBD7 under hypoxia, thus promoting HCC metastasis through tumor cell adhesion and EMT. Hypoxia in the tumour microenvironment influences the entire stages of hepatocellular carcinoma (HCC) metastasis with unarticulated mechanisms.^1^ Activation of hypoxia‐inducible factor (HIF) signalling promotes the invasion of tumour cells and infiltrating immune cells and naturally selects the tumour cells that survived under hypoxia.^2^ Previously, we have confirmed that BTBD7 in HCC microenvironment can promote epithelial‐mesenchymal transition (EMT) and stimulate angiogenesis in vitro and in vivo.^3^ However, BTBD7 does not be concerned about hypoxia‐associated particular mechanisms.

We observed increased BTBD7 mRNA and protein expression in HCC cells (MHCC97L and HCCML3) under hypoxia compared with normoxia condition (Figure 1A, B). The qPCR results of 78 paired HCC tissues and adjacent non‐tumorous tissues demonstrated that HIF‐1α mRNA was significantly overexpressed in HCC (Figure S1), suggesting that HIF‐1α was probably involved in hypoxia‐mediated BTBD7 upregulation, which was preliminarily confirmed by using the HIF‐1α activators (CoCl_2_ and DFO) or inhibitors (17‐AAG and Deguelin) (Figures 1C and S2) in HCC cells.

**FIGURE 1:**
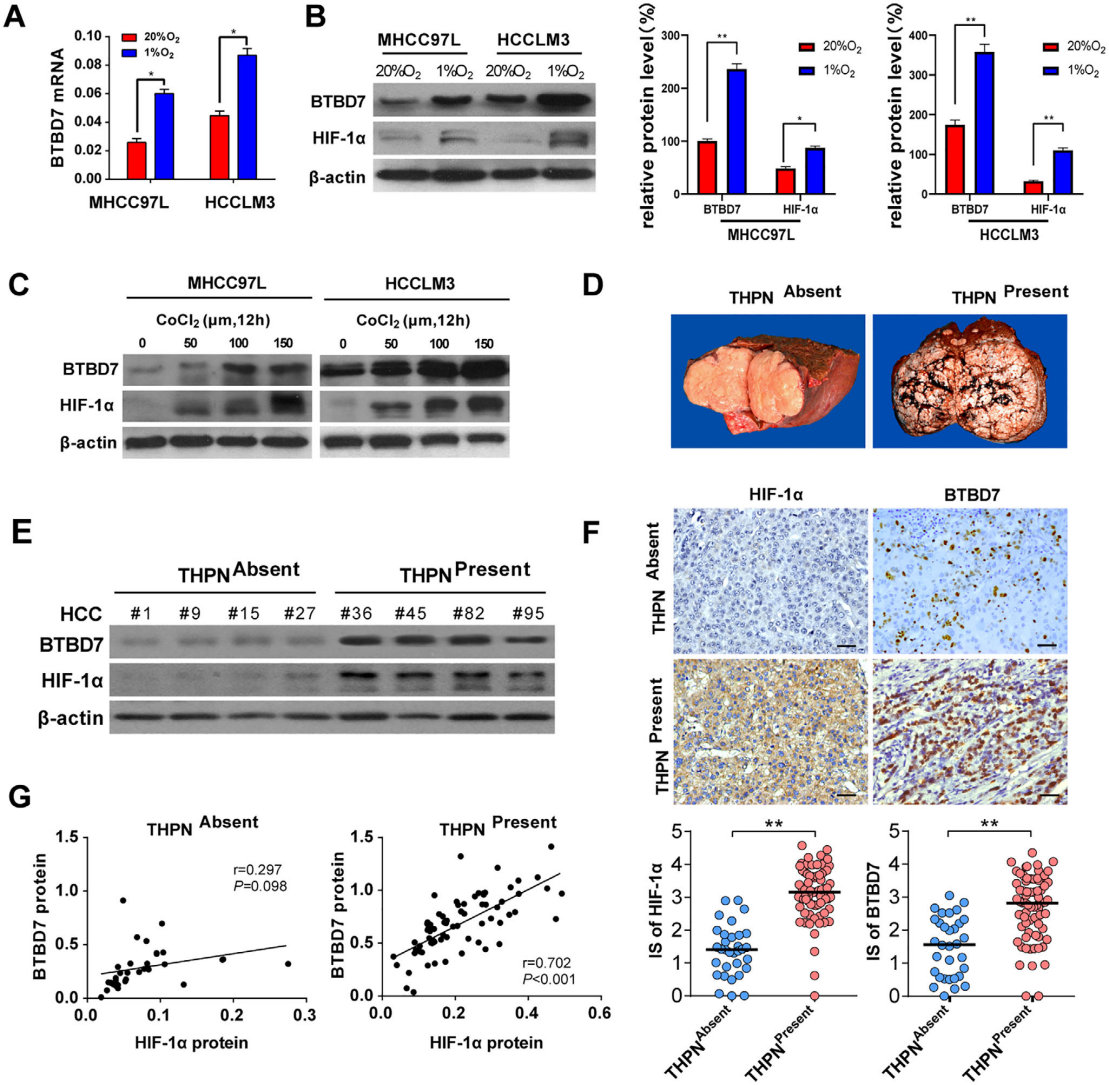
Hypoxia induces BTBD7 expression in HCC cells and tissues. (A) Relative mRNA expression of BTBD7 was detected by qPCR in MHCC97L and HCCLM3 cells cultured under hypoxia (1% O_2_) or normoxia (20% O_2_) for 24 h (*n* = 3, *p<0.05). (B) Western blot of BTBD7 and HIF‐1α in MHCC97L and HCCLM3 cells cultured under hypoxia (1% O_2_) or normoxia (20% O_2_) for 24 h (*n* = 3, *p<0.05, **p <0.01). (C) HIF‐1α activator (Cobalt chloride, CoCl_2_) induced upregulation of BTBD7 and HIF‐1α expression at the protein level. (D) In a training cohort of 104 paired of HCCs, the patients were divided into HCC tissues characterized with tumour haemorrhage plus necrosis (THPN^Present^) and without tumour haemorrhage plus necrosis (THPN^Absent^) groups according to the pathological diagnosis. (E) Western blot of BTBD7 and HIF‐1α from representative HCC tissue samples. BTBD7 and HIF‐1α expression were higher in THPN^Present^ group than THPN^Absent^ group. (F) Representative immunohistochemistry staining of HIF‐1α and BTBD7 in HCC specimen (scale bar = 50 μm). The immunohistochemical score (IS) of HIF‐1α and BTBD7 was significantly higher in THPN^Present^ group than that in THPN^Absent^ group (*n* = 32 in THPN^Absent^ group, *n* = 72 in THPN^Present^ group; **p<0.01). (G) A positive correlation was found between BTBD7 and HIF‐1α protein expression in THPN ^Present^ HCC samples

The correlation between HIF‐1α and BTBD7 expression in HCC was further investigated in a training cohort of 104 HCC patients using a western blot, showing higher expressions of HIF‐1α and BTBD7 in HCC tissues characterized with tumour haemorrhage plus necrosis (THPN) (Figure 1D, E). THPN subtype in HCC reflected much severer hypoxia level, higher incidence of intrahepatic metastasis and vessel invasion (Figure S3). Immunohistochemistry of HIF‐1α and BTBD7 also gave consistent results (Figure 1F). HIF‐1α protein positively correlates with BTBD7 protein expression in THPN ^Presence^ HCC tissue (Figure 1G). These data indicated that HIF‐1α might upregulate BTBD7, thus enhancing HCC metastasis.

We further clarified the role of HIF‐1α in regulating BTBD7 expression. Significantly, downregulated BTBD7 was detected when HIF‐1α was knocked down in hypoxic HCC cells (Figure 2A). Then, we investigated whether BTBD7 was a direct target of HIF‐1α. Bioinformatics analysis predicted that there were three potential hypoxia‐responsive elements (HREs) within a 5’‐flanking region 1‐kb region upstream of the transcriptional start site of BTBD7 (Figure 2B). Luciferase assay proved that the transcriptional activity of BTBD7 was abolished under hypoxia by HRE2‐mut (PGL3‐BTBD7‐581M) instead of HRE1‐mut (PGL3‐BTBD7‐1000M) and HRE3‐mut (PGL3‐BTBD7‐40M) (Figure 2C). Chromatin immunoprecipitation confirmed that HIF‐1α could directly bind to HRE2 elements in BTBD7 promoter under hypoxia (Figure 2D).

**FIGURE 2:**
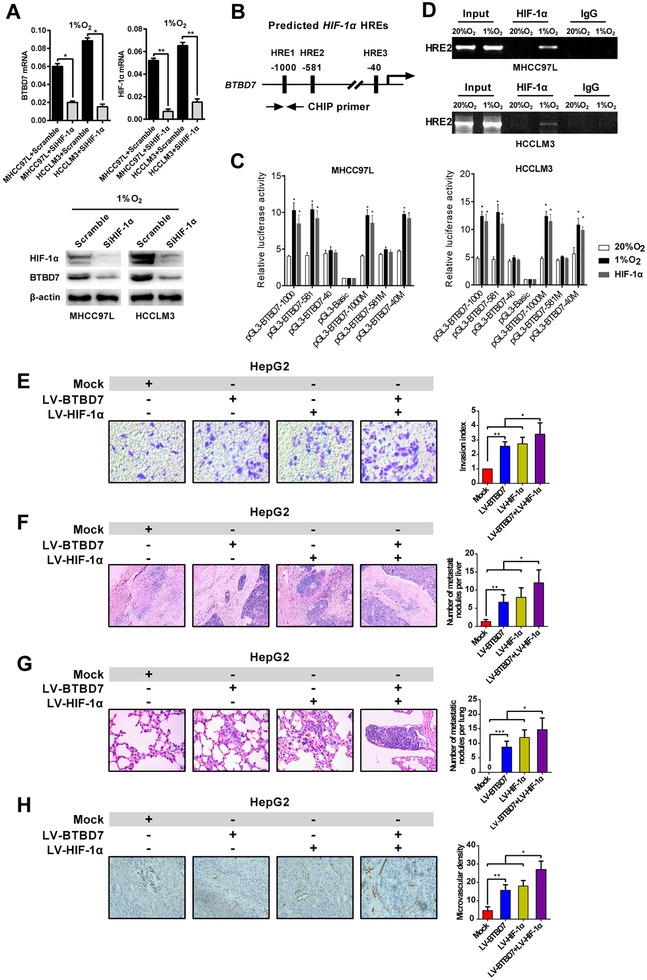
HIF‐1α directly mediates hypoxia‐induced BTBD7 expression and promotes metastasis and invasion of HCC cells in vitro and in vivo. (A) mRNA and protein levels of BTBD7 and HIF‐1α in MHCC‐97L and HCCLM3 cells under hypoxia (1% O_2_) when HIF‐1α was silenced (*n* = 3, *p<0.05, **p<0.01). (B) Schema shows a 5’upstream region of the BTBD7 gene with three consensus HREs of HIF‐1α. HRE1: PGL3‐BTBD7‐1000. HRE2: PGL3‐BTBD7‐581. HRE3: PGL3‐BTBD7‐40. The arrow means the chromatin immunoprecipitation (ChIP) primer of BTBD7 used for PCR. (C) Luciferase reporter assay of MHCC97L and HCCLM3 cells transfected with the luciferase reporter constructs was shown under hypoxia or transfected with LV‐HIF‐1α. Relative luciferase activities were expressed compared with the activity of the pGL3‐Basic (*n* = 3, bars indicate the mean ± SEM, *p<0.05). (D) ChIP assay was conducted with the antibody against HIF‐1α or control IgG in MHCC‐97L and HCCLM3 cells exposed to hypoxia (1% O_2_) or normoxia (20% O_2_) for 24 h. (E) Matrigel invasion assays were conducted to analyse the effect of BTBD7 and HIF‐1α on the invasion of HepG2 HCC cell lines (*n* = 3, *p<0.05, **p<0.01, scale bar = 50 μm). (F–H) HepG2 HCC cells overexpressing BTBD7, HIF‐1α or both were used to build the xenograft mouse model. Representative images of haematoxylin‐eosin staining of metastatic nodules in the liver (F) or lungs (G) were shown from each animal group. Representative images from tumour sample serial sections for CD34 staining were shown in (H) (*n* = 6, *p<0.05, **p<0.01, ***p<0.001, scale bar = 200 μm)

Next, we performed invasion assays to explore the roles of BTBD7 and HIF‐1α in HCC metastasis under hypoxia. The invasion capability of HCC cells was critically disrupted after the co‐knockdown of BTBD7 and HIF‐1α, and was moderately decreased when si‐BTBD7 or si‐HIF‐1α was transfected separately (Figure S4). Besides, the overexpression of BTBD7 or HIF‐1α enhanced the invasion activity of HepG2 cells and exhibited a combined effect when BTBD7 and HIF‐1α were co‐overexpressed (Figure 2E). To recapitulate this in vivo, we established HepG2 cells with stable over‐expressed BTBD7 or HIF‐1α or both, then built xenograft tumour‐bearing nude mice. The results showed that the most increased intrahepatic and pulmonary metastatic nodules and microvascular density were found in the co‐overexpressed group compared with other groups (Figures 2F–H).

Cell adhesion was measured after the overexpression of BTBD7 or HIF‐1α with the consideration of the crosstalk between HIF‐1α and the α5β1‐integrin signalling pathway in cell‐matrix adhesion.^4^ The cell adhesion to collagen, fibronectin and laminin increased 1.5‐ to 2.5‐fold (Figure 3A, B). Then, western blot was performed to identify the adhesion‐associated molecules regulated by BTBD7. We found elevated expression of α5β1‐integrin, fibronectin, p‐FAK, p‐Smad2, p‐STAT3, Ikk‐β and MMP‐9 in HepG2 cells that overexpressed BTBD7 or HIF‐1α or both (Figure 3C). The antibodies against α5‐integrin and β1‐integrin showed a synergistic effect with si‐BTBD7 in decreasing the HCC cell‐matrix adhesion (Figure 3D). Besides, the enhanced adhesion to collagen and laminin was blocked by an anti‐fibronectin antibody (Figure S5). These results indicated that BTBD7 and HIF‐1α could positively modulate HCC cell adhesion.

**FIGURE 3:**
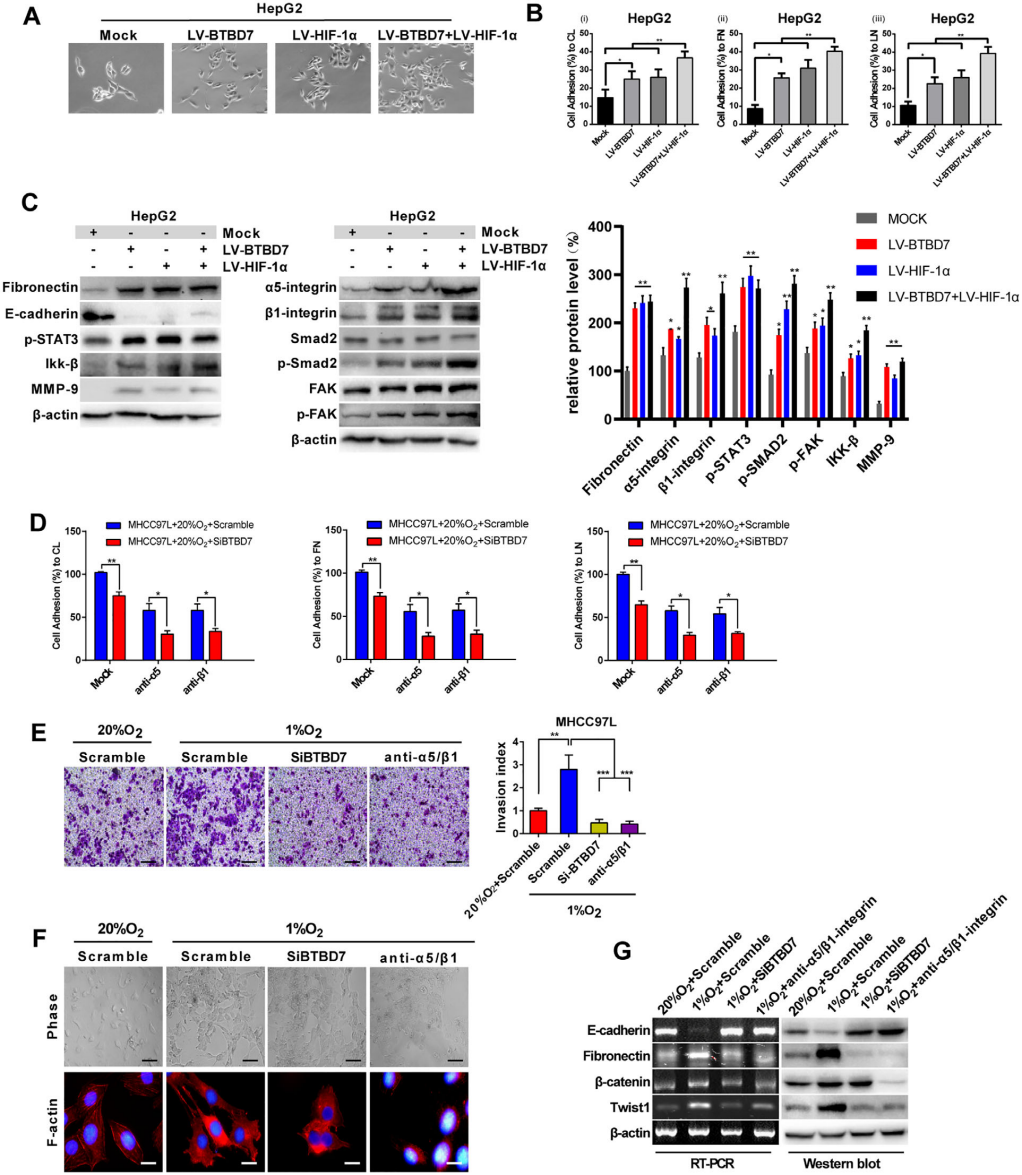
Effect of BTBD7/HIF‐1α expression on HCC adhesion and EMT process. (A) Phase‐contrast microphotographs of HepG2 cell morphology when BTBD7, HIF‐1α or both were transfected, respectively. (B) Cells were labelled with BCECF‐AM and detected for adhesion to 96‐well plates coated with collagen (i), fibronectin (ii) or laminin (iii), then cell adhesion assay was performed. Abbreviations: CL, collagen; FN, fibronectin; LN, laminin. (*n* = 3, *p<0.05, **p<0.01). (C) Western blot of adhesion‐associated proteins regulated by BTBD7 and HIF‐1α in HepG2 cells. The phosphorylation sites used for western blot were p‐smad2 (Ser465/467), p‐stat3 (Tyr705) and p‐FAK (Tyr397). (*n* = 3, *p<0.05 vs. Mock, **p<0.01 vs. Mock). (D) The antibodies against α5‐integrin and β1‐integrin showed synergistic effect with si‐BTBD7 in decreasing the HCC cell‐matrix adhesion (*n* = 3, *p<0.05, **p<0.01). (E) Matrigel invasion assays in MHCC‐97L cells after BTBD7 silence or anti‐α5β1‐integrin treatment under hypoxia (1% O_2_) (*n* = 3, **p<0.01, ***p<0.001, scale bar = 50 μm). (F) Morphology and F‐actin immunofluorescence of MHCC‐97L cultured under hypoxic conditions (1% O_2_) after BTBD7 silence or anti‐α5β1‐integrin treatment were shown by phase‐contrast microscopy (upper panel, scale bar = 50 μm) or fluorescence microscopy (lower panel, scale bar = 10 μm). F‐actin and DAPI are shown in red and blue, respectively. (G) RT‐PCR and western blot assays showed the upregulation of E‐cadherin expression and abolishment of fibronectin upregulation in MHCC97L cells after BTBD7 silence or anti‐α5β1‐integrin treatment under hypoxia (1% O_2_)

Both cell adhesion and EMT are induced by hypoxia to promote HCC metastasis.^5^, ^6^ Therefore, we explored whether BTBD7 played a significant role in hypoxia‐induced EMT through α5β1‐integrin. Either knockdown of BTBD7 or anti‐α5β1‐integrin significantly reduced invasion ability in MHCC97L cells induced by hypoxia (Figure 3E), inhibited the formation of typical pipe‐like structure within the Matrigel and decreased the in vitro F‐actin stress fibre formation induced by hypoxia in MHCC97L cells (Figure 3F). Western blot and qRT‐PCR assays showed the upregulation of E‐cadherin expression and abolishment of fibronectin and vimentin upregulation in MHCC97L cells under si‐BTBD7 or anti‐α5β1‐integrin (Figures 3G and S6). These results supported that activation of BTBD7 by HIF‐1α was required for hypoxia‐induced HCC cells EMT via the α5β1‐integrin pathway.

Given the central role of the HIF‐1α/BTBD7 axis in HCC adhesion and EMT, we chose Fasudil, the HIF‐1α inhibitor,^7^ as a potential therapeutic agent in BTBD7‐positive HCC. Fasudil treatment induced a dose‐dependent decrease expression of HIF‐1α and BTBD7 in the MHCC97L cells under hypoxia (Figure S7). Administration of Fasudil in HepG2‐LV‐BTBD7 xenograft tumour‐bearing nude mice obviously inhibited tumour growth (Figure 4A) and metastasis (Figure 4B, C). Meanwhile, the administration of Fasudil significantly downregulated the levels of BTBD7, HIF‐1α, TGF‐β1 and α5β1‐integrin and upregulated the level of E‐cadherin (Figure 4D).

**FIGURE 4:**
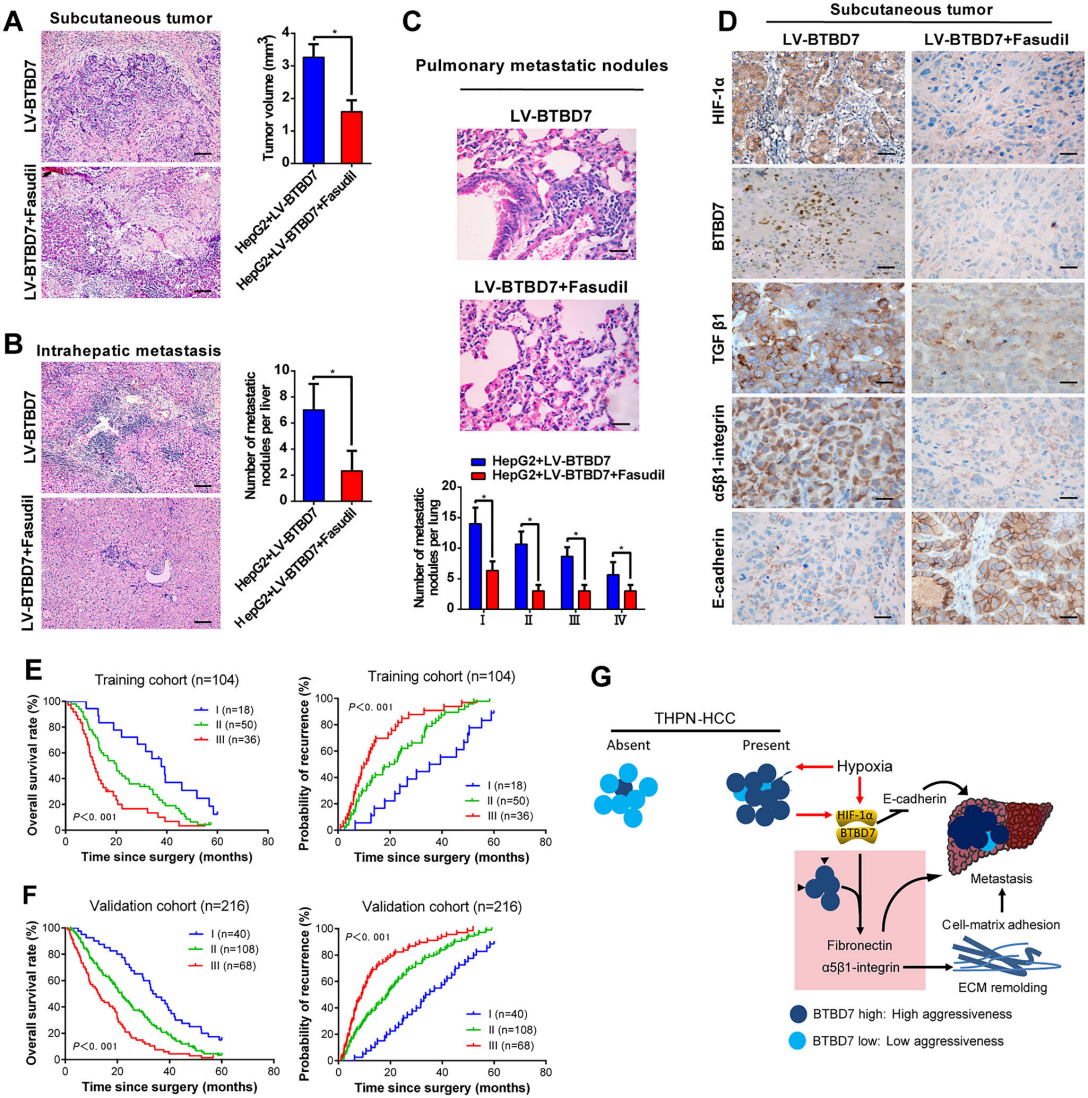
Pharmacological inhibition of HIF‐1α disrupts BTBD7/HIF‐1α‐induced HCC growth and metastasis in vivo and the prognostic value of HIF‐1α combined with BTBD7 in HCC patients. (A) Administration of Fasudil in HepG2‐LV‐BTBD7 xenograft tumour‐bearing nude mice obviously slowed down the growth of tumour. (B and C) Typical H&E images for the intrahepatic (B) and pulmonary metastatic nodules (C) between two groups in HCC mouse models (*n* = 6, *p<0.05, scale bar = 200 μm). (D) The effects of Fasudil on BTBD7, HIF‐1α, TGF‐β1, α5β1‐integrin and E‐cadherin expression in vivo (scale bar = 50 μm). (E) The prognostic roles of HIF‐1α combined with BTBD7 expression in OS and TTR of HCC patients in the training cohort. The patients are stratified into three groups. Group I, HIF‐1α^Low^/BTBD7^Low^; Group III, HIF‐1α^High^/BTBD7^High^; Group II, others. Abbreviations: OS, overall survival; TTR, time to recurrence. (F) Evaluation of HIF‐1α combined with BTBD7 expression in validation cohort as an independent predictor of poor clinical outcome in patients with HCC. Group stratification was the same as shown in (E). (G) An illustration of how HIF‐1α and BTBD7 were induced by the hypoxic tumour microenvironment and promoted HCC tumour cell adhesion and EMT with clinical prognostic and therapeutic significance

Univariate and multivariate analyses revealed that BTBD7 and HIF‐1α expression were independent predictors of cumulative overall survival (OS) and time to recurrence in the training cohort (Table S1). BTBD7 and HIF‐1α could act as a combined biomarker on the prognosis of HCC because three subgroups stratified according to BTBD7 and HIF‐1α showed a significant difference in OS and recurrence rates in both training and validation cohorts (Figure 4E, F). High expression of both BTBD7 and HIF‐1α indicated a poor prognosis.

In summary, this study is the first to elucidate that HIF‐1α and BTBD7 induced by hypoxic tumour microenvironment can promote HCC tumour cell adhesion and EMT with clinical prognostic and therapeutic significance (Figure 4G). TNPH may serve as a visualized and convenient phenotype to indicate high expression of HIF‐1α and BTBD7 for preliminary clinical screening.

## CONFLICT OF INTERESTS

The authors declare that they have no competing interests.

## Supporting information

Supporting information

Supporting information

figureS1

figureS2

figureS3

figureS4

figureS5

figureS6

figureS7

## References

[1] Yuen VW , Wong CC . Hypoxia‐inducible factors and innate immunity in liver cancer. J Clin Invest. 2020;130:5052‐5062. 32750043 10.1172/JCI137553 PMC7524494

[2] Ma Z , Xiang X , Li S , et al. Targeting hypoxia‐inducible factor‐1, for cancer treatment: recent advances in developing small‐molecule inhibitors from natural compounds. Semin Cancer Biol. 2020;28(20):30202–30209. 10.1016/j.semcancer.2020.09.011. 33002608

[3] Tao YM , Huang JL , Zeng S , et al. BTB/POZ domain‐containing protein 7: epithelial‐mesenchymal transition promoter and prognostic biomarker of hepatocellular carcinoma. Hepatology. 2013;57:2326–2337. 23325674 10.1002/hep.26268

[4] Li H , Ge C , Zhao FY , et al. Hypoxia‐inducible factor 1 alpha‐activated angiopoietin‐like protein 4 contributes to tumor metastasis via vascular cell adhesion molecule‐1/integrin beta1 signaling in human hepatocellular carcinoma. Hepatology. 2011;54(3):910–919. 21674552 10.1002/hep.24479

[5] Liu Z , Wang Y , Dou C , et al. Hypoxia‐induced up‐regulation of VASP promotes invasiveness and metastasis of hepatocellular carcinoma. Theranostics. 2018;8:4649–4663. 30279729 10.7150/thno.26789 PMC6160773

[6] Xu Z , Shen MX , Ma DZ , Wang LY , Zha XL . TGF‐beta1‐promoted epithelial‐to‐mesenchymal transformation and cell adhesion contribute to TGF‐beta1‐enhanced cell migration in SMMC‐7721 cells. Cell Res. 2003;13:343–350. 14672557 10.1038/sj.cr.7290179

[7] Xia Y , Cai X‐Y , Fan J‐Q , et al. Rho kinase inhibitor Fasudil suppresses the vasculogenic mimicry of B16 mouse melanoma cells both in vitro and in vivo. Mol Cancer Ther. 2015;14:1582–1590. 25934709 10.1158/1535-7163.MCT-14-0523

