# Supplementary material for: BTB/POZ domain‐containing protein 7/hypoxia‐inducible factor 1 alpha signalling axis modulates hepatocellular carcinoma metastasis
Source: Clin Transl Med. 2021 Oct 12;11(10):e556. doi: 10.1002/ctm2.556 (PMC8506631; doi:10.1002/ctm2.556)
Supplement: Supplementary file 1 — Supporting information [file CTM2-11-e556-s004.docx]

**Materials & Methods**

***Cell Lines.*** The cell line HepG2 was purchased from American Type Culture Collection (ATCC, Rockville, MD); MHCC97L and HCCLM3 cell lines were obtained from the Liver Cancer Institute of Zhongshan Hospital, Shanghai, Fudan University, China. All cell lines were cultured in DMEM (Dulbecco’s modified Eagle’s medium) (Sigma) supplemented with 10% FBS (fetal bovine serum) (Hyclone) and incubated at 37°C with 5% CO_2_. Hypoxic conditions (1% O_2_) experiments were performed by using a NEPCO 1000 hypoxia incubator (Warrensburg, NY). Cobalt chloride (CoCl_2_) and Deferoxamine mesylate (DFO) (Sigma) was used to mimic the effects of hypoxia at 1% O_2_ [1,2]. All HCC cell lines were propagated under hypoxia condition (1% O_2_ or of the treatment with hypoxic agent) as previously described [3,4].

***HCC Specimens.*** For the acquisition of clinical samples, informed consent was signed by each patient with HCC at the day before surgery. The whole protocol was approved by the Ethics Committee of Xiangya Hospital, China (201303034). Two independent HCC cohorts were enrolled in this study. The clinical outcomes of HCC participants are listed in Supplementary Table S2. The termination time of patient follow-up was 31 January 2016. The median follow-up time was 36.5 months (range from 2 to 60 months).

***Quantitative Real-Time RT-PCR (qRT-PCR).*** The procedures of SYBR Green fluorescent-based qRT-PCR were performed as previously described [3]. Oligonucleotide sequences are listed in Supplementary Table S3.

***Western blot, Immunohistochemistry (IHC), and Immunofluorescence (IF) Staining.*** Western blot and IHC were performed as previously described [3]. The antibodies are listed in Supplementary Table S4. For IF staining, we use phosphate-buffered saline (PBS, pH 7.4) to rinse HCC cells, then fixed them with 3% paraformaldehyde. 0.1% Triton X-100 was used to improve the penetration, then the cells were incubated with 5% bovine serum albumin in PBST (PBS+ 0.05% Tween 20) for 30 mins. With the steps of the incubation of first antibody, three times washing with PSB, and incubation of secondary antibody and DAPI (Invitrogen), the images were obtained by using fluorescence microscope.

***Gene Silencing and Overexpression.*** Small interfering RNAs (siRNAs) of BTBD7 and HIF-1α, expression plasmids of BTBD7 and HIF-1α were as previously described and the procedures of gene silencing and overexpression were carried out as previously described [3,5].

***Blocking Studies***. We incubated the MHCC97L cells for 60 minutes with 10μg/ml function blocking anti-integrin α5 (Clone P1D6; BioLegend, US) or anti-integrin β1 (clone N29; Millipore, Germany). Cell culture medium was used for MHCC97L cells incubation in control group. Then these MHCC97L cells were subjected to the cell adhesion assay.

***Cell adhesion assay and Invasion assays.*** For the cell adhesion assay, HepG2 and MHCC97L cell attachment to fibronectin, collagen, and laminin was performed as described in a published paper [6]. Briefly, HCC cells labeled by the fluorescent dye BCECF-AM (Calbiochem-Novabiochem Co.) were added to 96-well plates coated by ligand proteins. Cells remaining attached to the 96-well plate were evaluated after several steps of washing. The invasion assay was performed as previously described [3].

***Chromatin immunoprecipitation (ChIP).*** ChIP assays were performed to analyze whether HIF-1α transactivates BTBD7 promoter. MHCC97L and HCCLM3 cells were cultured under hypoxic or normoxic conditions for 24 hours before DNA-protein cross-linking in PBS containing 1% formaldehyde for ten minutes. Next, the ChIP procedure was conducted with the Imprint Chromatin Immunoprecipitation Kit (Sigma, St Louis, MO) using the manufacturer’s recommended protocol. The anti-HIF-1α antibodies (Cell Signaling Technology) or control anti-IgG antibodies (Sigma) were used. Next, precipitated DNA was purified first, then these DNA was used as a template for RT-PCR together with primers flanking putative HIF binding sites located on the BTBD7 promoter (Supplementary Table S3)

***Luciferase reporter assays.*** Cells seeded in 96-well pates were transfected with promoter reporter plasmids (45ng) together with HIF-1α plasmids (150ng) or under hypoxic (1% O_2_) or under normoxic conditions (20% O_2_). Then the cell extracts were used to perform the Dual-luciferase reporter assay according to the instructions from the manufacturer (Promega, USA). The Renilla luciferase activity was used for normalization.

***Animal Model Studies.*** All animal assays in this study were performed according to the protocols approved by the Animal Care and Use Committee at Central South University, China (201303033). HepG2-LV-BTBD7, HepG2-LV-HIF-1α and HepG2-LV-BTBD7-HIF-1α xenograft tumor-bearing nude mice model was built in accordance with an existing protocol [3]. For the administration of Fasudil in vivo, the tumor-bearing nude mice were randomly classified (at the time point that the tumor diameter reached to about 5 mm) into vehicle group (receive PBS intraperitoneally 50mg/kg daily for 14 days) and Fasudil group (receive Fasudil intraperitoneally 50mg/kg daily for 14 days).

***Statistical Analysis.*** Statistical analysis was performed using SPSS 18.0 software (SPSS, Chicago, IL). Quantitative values are presented as mean ± SEM. Paired t tests and student’s tests were used for paired and unpaired continuous data respectively. Spearman’s correlation coefficient (*r*) was used to access the correlation of protein levels between BTBD7 and HIF-1α genes. χ2 test was applied for categorical data. The cumulative time to recurrence (TTR) and overall survival (OS) were evaluated using the Kaplan-Meier method and the Log-rank test. Cox proportional hazards regression model was used to determine if BTBD7 expression combined with HIF-1α is an independent prognostic indicator. *P* < 0.05 was considered to be statistically significant.

**Supplementary Figures and Tables**


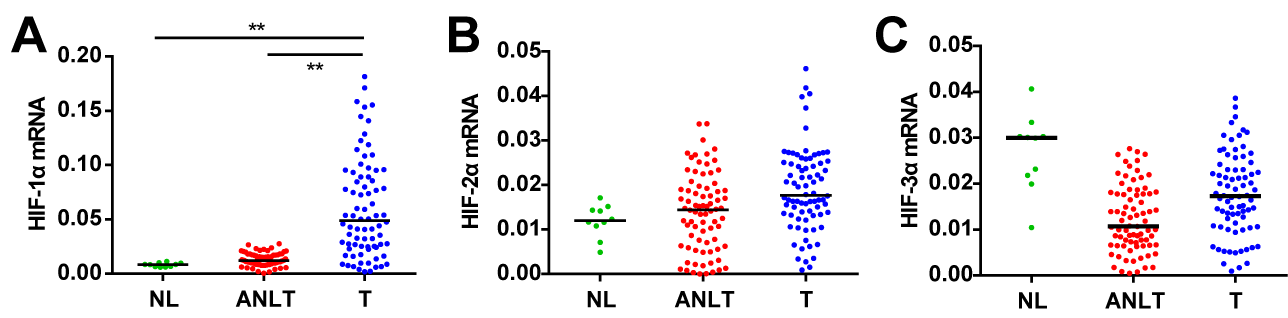


**Supplementary Figure S1. qRT-PCR analysis of the expressions of HIFs (HIF-1α/HIF-2α/HIF-3α) in the 78 paired HCC samples. (A-C)** HIF-1α was most substantially overexpressed among all three HIFs including HIF-1α (A), HIF-2α (B), HIF-3α (C) in HCC tissues. NL (n=10), ANLT (n=78) and T (n=78) represent normal liver tissue, adjacent non-tumorous liver tissue, tumor tissue, respectively. ** *p*<0.01.


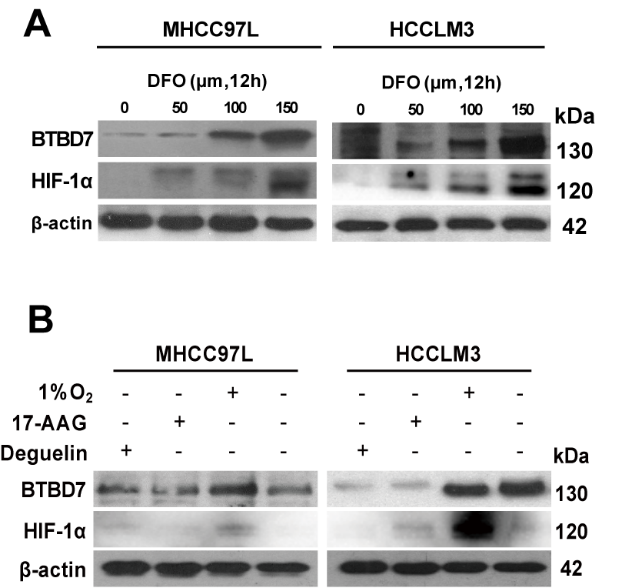


**Supplementary Figure S2. BTBD7 expression altered in HCC cells after treatment with HIF-1α activators or inhibitors.** (A-B) Western blot analysis of BTBD7 and HIF-1α levels in HCC cells (MHCC97-L and HCCLM3) after treated with HIF-1α activators (Deferoxamine mesylate, DFO) (A) or inhibitors (17-AAG and Deguelin) (B) for 12h.


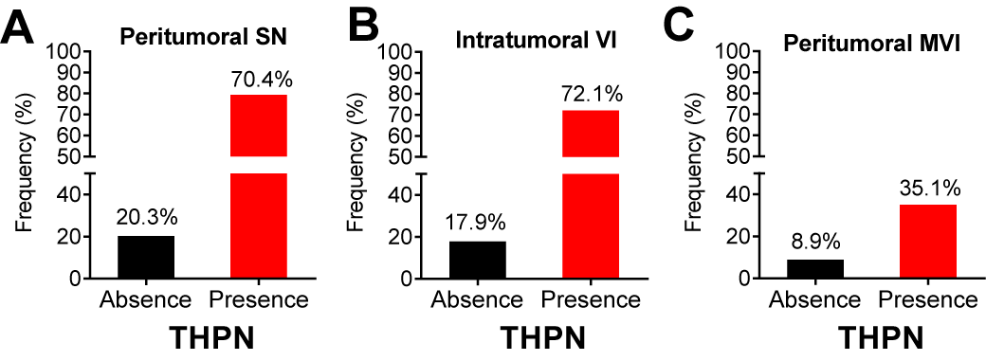


**Supplementary Figure S3. Intrahepatic metastases were more frequently observed in HCC specimens with tumor hemorrhage plus necrosis (THPN).** (A) Peritumoral SN between THPN^Absence^ and THPN^Presence^ group. (B) Intratumoral VI between THPN^Absence^ and THPN^Presence^ group. (C) Peritumoral MVI between THPN^Absence^ and THPN^Presence^ group. SN: satellite nodules, VI: vessel invasion, MVI: microvessel invasion.


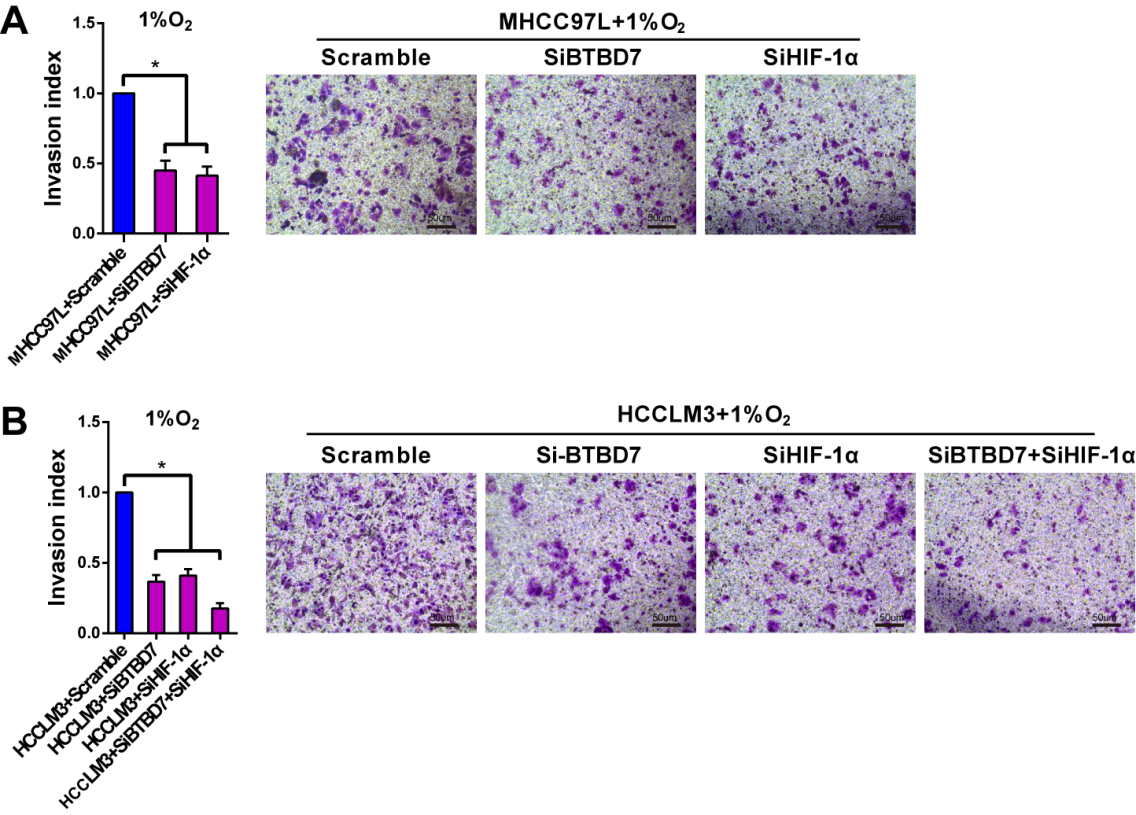


**Supplementary Figure S4. HIF-1α functionally cooperates with BTBD7 in promoting HCC cell invasion under hypoxic conditions (1% O_2_).** (A-B) Matrigel invasion assays showing invasion capability inhibition in HCC cells with knockdown of HIF-1α or BTBD7 (A) or both (B). Representative images are shown (n = 3 in each group, * *p*<0.05).


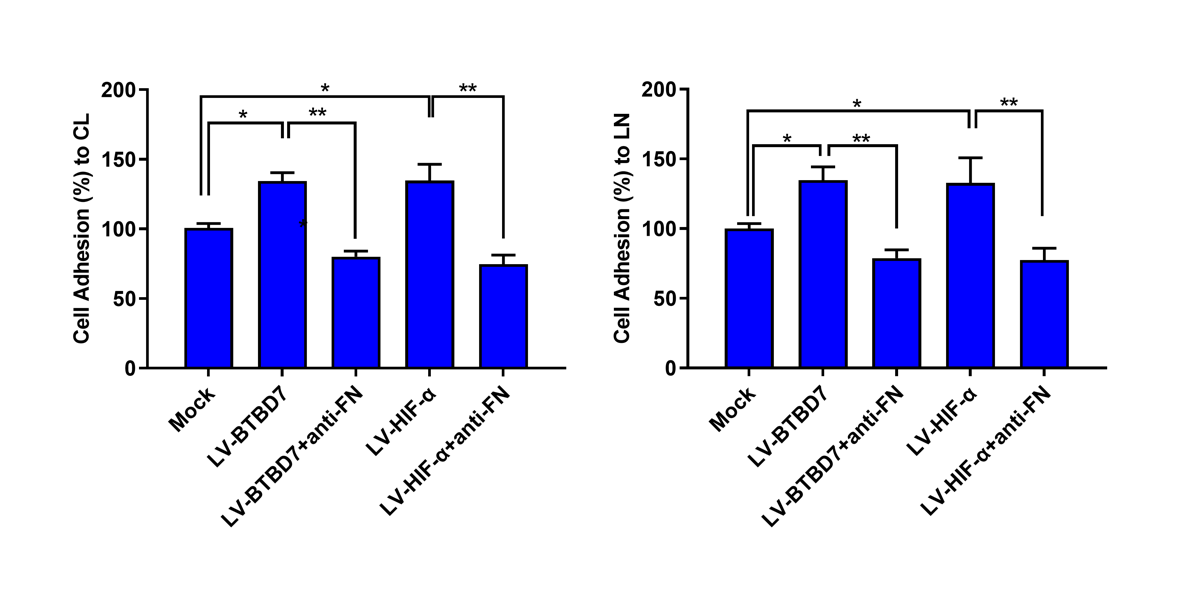


**Supplementary Figure S5. MHCC97L cells adhesion to CL and LN with the use of anti-FN**. After overexpression of BTBD7 or HIF-1α, MHCC97L cells were treated with anti-FN, and then cell adhesion assay was performed. CL, collagen; FN, fibronectin; LN, laminin. (n = 3, * p <0.05, ** p<0.01.)


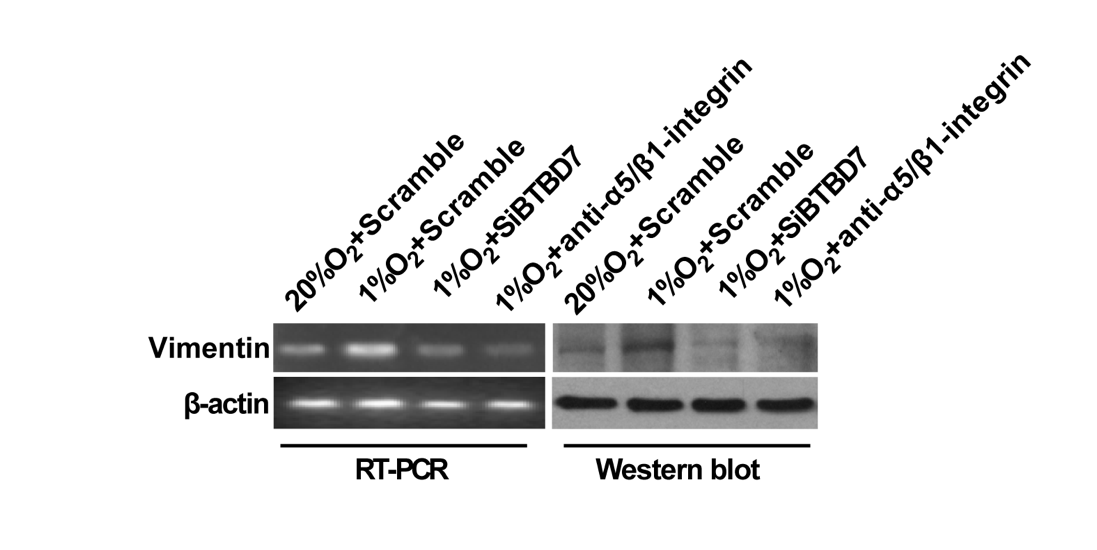


**Supplementary Figure S6.** Western blot and qRT-PCR assays to test the vimentin level in MHCC97L cells treated with si-BTBD7 or anti-α5β1-integrin under normoxic and hypoxic conditions.


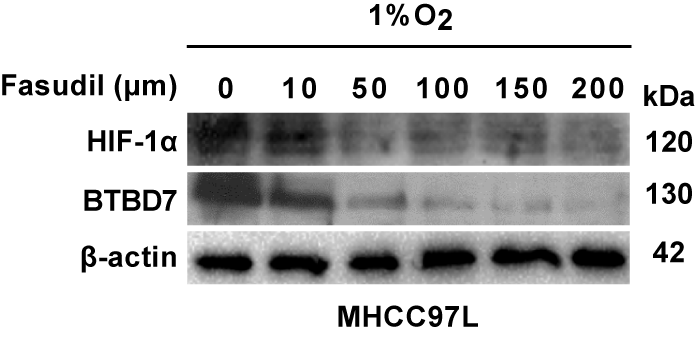


**Supplementary Figure S7. Effect of Fasudil treatment on HIF-1α and BTBD7 expression in MHCC97L cells under hypoxic conditions (1%O_2_).** Downregulation of HIF-1α and BTBD7 protein levels was dose-dependently induced by Fasudil treatment.

**Supplementary Table S1. Univariate and Multivariate analyses of prognostic factors with TTR and OS in HCC patients (n=104)**

|  | **TTR** |  | **OS** |  |
| --- | --- | --- | --- | --- |
| **Variable** | **HR(95% CI)** | ***P*** | **HR(95% CI)** | ***P*** |
| **Univariate analysis**† |  |  |  |  |
| Gender (male vs. female) | 1.132 (0.994-1.354) | 0.086 | 1.078 (0.654-1.215) | 0.352 |
| Age, years (> 60 vs. ≤ 60) | 1.062 (0.745-1.321) | 0.234 | 1.134 (0.952 -1.369) | 0.081 |
| HBsAg (positive vs negative) | 1.142 (0.874-1.562) | 0.124 | 1.201 (0.899-1.562) | 0.116 |
| Albumin, g/L(≤35 vs. >35) | 1.097 (0.738-1.546) | 0.316 | 1.195 (0.972-1.463) | 0.064 |
| Child-Pugh classification (B vs. A) | 1.321 (1.057-1.875) | **0.040** | 1.348 (1.094-1.845) | **0.032** |
| Liver cirrhosis (presence vs absence) | 1.246 (1.013-2.533) | **0.017** | 1.296 (1.052-1.748) | **0.041** |
| Serum AFP level, ng/mL (> 20 vs. ≤ 20) | 1.725 (1.116-2.479) | **0.009** | 1.872 (1.316-2.764) | **0.009** |
| Tumor diameter, cm (> 5 vs. ≤ 5) | 1.418 (1.072-2.161) | **0.025** | 1.536 (1.132-2.567) | **0.013** |
| Tumor number (multiple vs. single) | 1.627 (1.139-2.754) | **0.003** | 2.167 (1.349-3.854) | **0.002** |
| Tumor encapsulation(none vs. complete) | 1.146 (0.924-1.737) | 0.064 | 1.472 (1.071-2.488) | **0.029** |
| Venous invasion (presence vs. absence) | 2.534 (1.476- 4.465) | **0.001** | 2.876 (1.326-5.241) | **<0.0001** |
| Tumor differentiation (III/IV vs. I/II ) | 1.163 (1.024-1.682) | **0.038** | 1.206 (0.944-2.218) | 0.075 |
| Satellite nodules (presence vs. absence) | 1.523 (1.179-2.164) | **0.012** | 1.351 (1.099-2.942) | **0.036** |
| THPN (presence vs. absence) | 1.841(1.058-3.617) | **0.010** | 1.518(1.102-3.477) | **0.008** |
| BCLC stage (B + C vs. A) | 1.312 (1.054-1.938) | **0.030** | 1.456 (1.113-2.326) | **0.015** |
| TNM stage (II/III vs. I) | 1.456 (1.137-2.695) | **0.007** | 1.692 (1.225-3.641) | **0.006** |
| BTBD7 expression level (high vs. low) | 2.994(1.785- 6.843) | **<0.0001** | 2.574 (1.263-5.346) | **0.001** |
| HIF-1α expression level (low vs. high) | 3.156 (1.359 -7.457) | **<0.0001** | 2.892 (1.931-7.432) | **<0.0001** |
| Combination of BTBD7 and HIF-1α^*^ |  |  |  |  |
| II vs I | 2.245 (1.524-5.762) | **0.002** | 1.925 (1.467-3.664) | **0.004** |
| III vs I | 8.513 (2.687-11.436) | **<0.0001** | 7.543 (3.124-9.876) | **<0.0001** |
| III vs II | 4.927 (2.232-8.421) | **<0.0001** | 3.769 (1.964-7.653) | **<0.0001** |
| **Multivariate analysis**† |  |  |  |  |
| Gender (male vs. female) | NA |  | NA |  |
| Age, years (> 60 vs. ≤ 60) | NA |  | NA |  |
| HBsAg (positive vs negative) | NA |  | NA |  |
| Albumin, g/L(≤35 vs. >35) | NA |  | NA |  |
| Child-Pugh classification (B vs. A) | 1.106 (0.746-1.564) | NS | 1.132 (0.972-1.546) | NS |
| Liver cirrhosis (presence vs absence) | 1.087 (0.877-1.654) | NS | 1.079 (0.833-1.364) | NS |
| Serum AFP level, ng/mL (> 20 vs. ≤ 20) | 1.241 (0.964-1.934) | NS | 1.248 (0.864-1.612) | NS |
| Tumor diameter, cm (> 5 vs. ≤ 5) | 1.197 (0.918-1.862) | NS | 1.114 (0.926-1.742) | NS |
| Tumor number (multiple vs. single) | 1.264 (1.085-2.124) | **0.033** | 1.543 (1.138-2.863) | **0.016** |
| Tumor encapsulation(none vs. complete) | NA |  | 1.207 (0.768-1.763) | NS |
| Venous invasion (presence vs. absence) | 2.261 (1.354-3.246) | **0.005** | 1.942 (1.365-4.533) | **0.005** |
| Tumor differentiation (III/IV vs. I/II ) | 1.132 (0.906-1.463) | NS | NA |  |
| Satellite nodules (presence vs. absence) | 1.796 (1.267-2.946) | **0.009** | 1.138 (1.016-2.564) | **0.042** |
| THPN (presence vs. absence) | 1.658(1.097-3.416) | **0.015** | 1.715(1.231-4.526) | **0.002** |
| BCLC stage (B + C vs. A) | 1.624 (1.142-2.843) | **0.021** | 1.875 (1.284-3.146) | **0.024** |
| TNM stage (II/III vs. I) | 1.724 (1.236-3.221) | **0.011** | 1.921 (1.354-4.275) | **0.008** |
| BTBD7 expression level (high vs. low) | 2.669 (1.368-5.945) | **<0.0001** | 2.274 (1.645-5.021) | **0.003** |
| HIF-1α expression level (low vs. high) | 3.421 (1.647-9.234) | **<0.0001** | 3.041 (1.743-8.847) | **<0.0001** |
| Combination of BTBD7 and HIF-1α^*^ |  |  |  |  |
| II vs I | 1.876 (1.246-4.547) | **<0.0001** | 1.795 (1.421-3.254) | **0.013** |
| III vs I | 7.546 (2.543-9.969) | **<0.0001** | 6.742 (2.305-8.246) | **<0.0001** |
| III vs II | 4.125 (1.946-7.243) | **<0.0001** | 3.214 (1.675-7.264) | **<0.0001** |

*:Ⅰ, BTBD7^Low^/HIF-1α^Low^; Ⅱ, BTBD7^High^/HIF-1α^Low^ and BTBD7^Low^/BTBD7^High^; Ⅲ, BTBD7^High^/HIF-1α^High^.

Abbreviations: HCC, hepatocellular carcinoma; TTR, time to recurrence; OS, overall survival; HR, hazard ratio; CI, confidential interval; NA, not adopted; HBsAg, hepatitis B surface antigen; AFP, α-fetoprotein; BCLC, Barcelona clinic liver cancer; THPN, tumor hemorrhage plus necrosis; TNM, tumor-node-metastasis, unless otherwise indicated. Significant difference is shown in bold.

† Cox proportional hazards regression

**Supplementary Table S2. The clinical outcomes of 320 HCC patients in training cohort and validation cohort.**

|  | Training cohort  (104 cases) | Validation cohort  (216 cases) |
| --- | --- | --- |
| Follow-up time(months) |  |  |
| Range | 2.0-60.0 | 2.0-60.0 |
| Median | 37.2 | 35.7 |
| Time to recurrence (months)# |  |  |
| Range | 1.0-60.0 | 1.0-60.0 |
| Median | 18.0 | 22.1 |
| Overall survival time (months) * |  |  |
| Range | 1.0-60.0 | 1.0-60.0 |
| Median | 23.5 | 24.6 |
| BCLC stage |  |  |
| A | 45 (43.3%) | 101 (46.8%) |
| B | 31 (29.8%) | 75 (34.7%) |
| C | 28 (26.9%) | 40 (18.5%) |
| TNM stage |  |  |
| Ⅰ | 53 (51.0%) | 138 (63.9%) |
| Ⅱ | 35 (33.7%) | 65 (30.1%) |
| Ⅲ | 16 (15.3%) | 13 (6.0%) |

# Time to recurrence (TTR) is calculated from the HCC resection to the first radiological evidence of recurrence. Patients with death in the absence of recurrence are censored.

* Overall survival (OS) is defined as the time interval between HCC surgery and death or the last observation. The data are censored at the last follow-up for living patients.

**Supplementary Table S3：Primer sequences used in this study.**

| **Primer name and sequences** |
| --- |
| **Primers for qRT-PCR:** |
| BTBD7 sense: 5’- agtcaaatgcctggttacgg -3’ |
| antisense: 5’- tgtctggcacattggacatt-3’ |
| HIF-1α sense: 5’-CCAGTTAGGTTCCTTCGATCAGT-3’ |
| antisense: 5’-TTTGAGGACTTGCGCTTTCA-3’  HIF-2α sense: 5’-AATCCGAGCAGTGGAGTCAT-3’  antisense: 5’-ACGTGCCATCAGACCCTCTT-3’  HIF-3α sense: 5’-CCTGGACATGAAGTTCACCTACTG-3’  antisense: 5’-GGAAGCGATACTGCCCTGTTA-3’ |
| E-cadherin sense: 5’-AGCCCCGCCTTATGATTCTCTG-3’ |
| antisense: 5’-TGCCCCATTCGTTCAAGTAGTCAT-3’ |
| β-catenin sense: 5’-ATGGAACCAGAGAAAAGC-3’ |
| antisense: 5’-CGGACTGTGTGATTGGTTCGA-3’ |
| Fibronectin sense: 5’- TGCATAGGGGATGATTTGTA-3’ |
| antisense: 5’- ATGACGATGGGAAGACCTAC-3’ |
| Twist1 sense: 5’-CGACGACAGCCTGAGCAACA-3’ |
| antisense: 5’-CCACAGCCCGCAGACTTCTT-3’  Vimentin sense: 5’-GACGCCATCAACACCGAGTT-3’  antisense: 5’-CTTTGTCGTTGGTTAGCTGGT-3’ |
| GAPDH sense: 5’-tgtctggcacattggacatt-3’ |
| antisense: 5’-GCACCGTCAAGGCTGAGAAC-3’ |
| **Primers for BTBD7 SiRNA:** |
| Control sense: 5’-GTGCGAATTGAACACATCTTA-3’ |
| antisense: 5’-CAAAAAGTGCGAATTGAACACA-3’ |
| BTBD7 siRNA-1 sense: 5’-CCACATTTAAAGGACTGTAT-3’ |
| antisense: 5’-CCCAATCCATAACCAACTCAA-3’ |
| BTBD7 siRNA-2 sense: 5’-CCCAATCCATAACCAACTCAA-3’ |
| antisense: 5’-CAAAAACCCAATCCATAACCAA-3’ |
| **Primers for HIF-1α SiRNA:** |
| Control sense: 5’-UUCUCCGAACGUGUCACGUTT-3’ |
| antisense: 5’-ACGUGACACGUUCGGAGAATT-3’ |
| HIF-1α siRNA-1 sense: 5’- GAAGGAACCUGAUGCUUUATT-3’ |
| antisense: 5’- UAAAGCAUCAGGUUCCUUCTT-3’ |
| HIF-1α siRNA-2 sense: 5’-CGAUGGAAGCACUAGACAATT-3’ |
| antisense: 5’-UUGUCUAGUGCUUCCAUCGGA-3’ |
| **Primers for HIF-1α expression:** |
| HIF-1α sense: 5’- GTACCCTAACTAGCCGAGGAAGAA-3’ |
| antisense: 5’- CTGAGGTTGGTTACTGTTGGTATCA-3’ |
| HIF-1α promoter-3-Mut sense: 5’-GCGGCGTGGGCGGGGACTTGCC-3’ |
| antisense: 5’-GGCAAGTCCCCGCCCACGCCGC-3’ |
| **Primers for BTBD7 promoter construct:** |
| BDBT7_1000 sense: 5’- ACGTCGGTACCTCACTGGGTTCAGAATGCCTGGG-3’ |
| antisense: 5’- CAGCAAAGATCTCCTCTCCTGTCAGTGGCTCAGGCTC-3’ |
| BDBT7_581 sense: 5’- GAAAACACACAGTCGCCAGCTGGGAGAGCCAAGTC-3’ |
| antisense: 5’- GACTTGGCTCTCCCAGCTGGCGACTGTGTGTTTTC-3’ |
| BDBT7_40 sense: 5’- CCGCTCAGGGTCAGCTGGCCCGACGTCG-3’ |
| antisense: 5’-CGACGTCGGGCCAGCTGACCCTGAGCGG -3’ |
| **Primers used for ChIP-PCR** |
| HRE2 of BTBD7 promoter sense: 5’- GCTAAACACAGACGAGCACGTG-3’ |
| antisense: 5’-TCACCTGAGGTGGAGGCGGGTT -3’ |

**Supplementary Table S4：The types, dilutions and sources of antibodies used for western blot and immunohistochemical analysis.**

| Antibody |  | Western blot | IHC | Source -Cat. Number |
| --- | --- | --- | --- | --- |
| BTBD7 |  | 1:1000 | 1:1000 | Novus Biologicals (Cat. No.NBP1-62182) |
| HIF-1α |  | 1:1000 | 1:800 | Cell Signaling Technology (Cat. No. #79233) |
| E-cadherin |  | 1:2000 | 1:1000 | Abcam (cat. No. ab15148 ) |
| Fibronectin |  | 1:1000 | **—** | Abcam (Cat. No. ab23750 ) |
| Twist1 |  | 1:2000 | **—** | Novus Biologicals (Cat. No. NB110-91359) |
| Vimentin |  | 1:1000 | **—** | Cell Signaling Technology (Cat. No. #5741) |
| beta-catenin |  | 1:200 | **—** | Abcam (Cat. No. ab16051 ) |
| Smad2 |  | 1:1000 | **—** | Cell Signaling Technology (Cat. No. #5339) |
| p-Smad2 |  | 1:1000 | **—** | Cell Signaling Technology (Cat. No. #3108) |
| STAT3 |  | 1:1000 | **—** | Cell Signaling Technology (Cat. No. #9139) |
| p-STAT3 |  | 1:2000 | **—** | Cell Signaling Technology (Cat. No. #9145) |
| Ikk-β |  | 1:1000 | **—** | Cell Signaling Technology (Cat. No. #8943) |
| α5-integrin |  | 1:1000 | 1:1000 | Cell Signaling Technology (Cat. No. #4705) |
| β1-integrin |  | 1:1000 | 1:1000 | Cell Signaling Technology (Cat. No. #4706) |
| FAK |  | 1:1000 | **—** | Cell Signaling Technology (Cat. No. #3285) |
| p-FAK |  | 1:1000 | **—** | Cell Signaling Technology (Cat. No. #8556) |
| TGF-β1 |  | **—** | 1:1000 | R&D Systems, Minneapolis, MN |
| CD34 |  | **—** | 1:1000 | Novus Biologicals (Cat. No. NBP1-44703) |
| MMP-9 |  | 1:1000 | **—** | Abcam (Cat. No. ab38898) |

**References**

1. Hallez, C.; Li, X.; Suspène, R.; Thiers, V.; Bouzidi, M.S.; C, M.D.; Lucansky, V.; Wain-Hobson, S.; Gaudin, R.; Vartanian, J.P. Hypoxia-induced human deoxyribonuclease I is a cellular restriction factor of hepatitis B virus. *Nat Microbiol* **2019**, *4*, 1196-1207, doi:10.1038/s41564-019-0405-x.

2. Wang, M.; Zhao, X.; Zhu, D.; Liu, T.; Liang, X.; Liu, F.; Zhang, Y.; Dong, X.; Sun, B. HIF-1α promoted vasculogenic mimicry formation in hepatocellular carcinoma through LOXL2 up-regulation in hypoxic tumor microenvironment. *J Exp Clin Cancer Res* **2017**, *36*, 60, doi:10.1186/s13046-017-0533-1.

3. Tao, Y.M.; Huang, J.L.; Zeng, S.; Zhang, S.; Fan, X.G.; Wang, Z.M.; Yang, H.X.; Yuan, X.H.; Wang, P.; Wu, F., et al. BTB/POZ domain-containing protein 7: epithelial-mesenchymal transition promoter and prognostic biomarker of hepatocellular carcinoma. *Hepatology* **2013**, *57*, 2326-2337, doi:10.1002/hep.26268.

4. Tao, Y.; Hu, K.; Tan, F.; Zhang, S.; Zhou, M.; Luo, J.; Wang, Z. SH3-domain binding protein 1 in the tumor microenvironment promotes hepatocellular carcinoma metastasis through WAVE2 pathway. *Oncotarget* **2016**, *7*, 18356-18370, doi:10.18632/oncotarget.7786.

5. Huang, Y.; Tao, Y.; Hu, K.; Lin, F.; Li, X.; Feng, T.; Wang, Z.-M. Hypoxia-induced NIPP1 activation enhances metastatic potential and predicts poor prognosis in hepatocellular carcinoma. *Tumor Biology* **2016**, *37*, 14903-14914.

6. Govaere, O.; Petz, M.; Wouters, J.; Vandewynckel, Y.P.; Scott, E.J.; Topal, B.; Nevens, F.; Verslype, C.; Anstee, Q.M.; Van Vlierberghe, H., et al. The PDGFRα-laminin B1-keratin 19 cascade drives tumor progression at the invasive front of human hepatocellular carcinoma. *Oncogene* **2017**, *36*, 6605-6616, doi:10.1038/onc.2017.260.
