# Supplementary figures and images for: BTB/POZ domain‐containing protein 7/hypoxia‐inducible factor 1 alpha signalling axis modulates hepatocellular carcinoma metastasis
Source: Clin Transl Med. 2021 Oct 12;11(10):e556. doi: 10.1002/ctm2.556 (PMC8506631; doi:10.1002/ctm2.556)

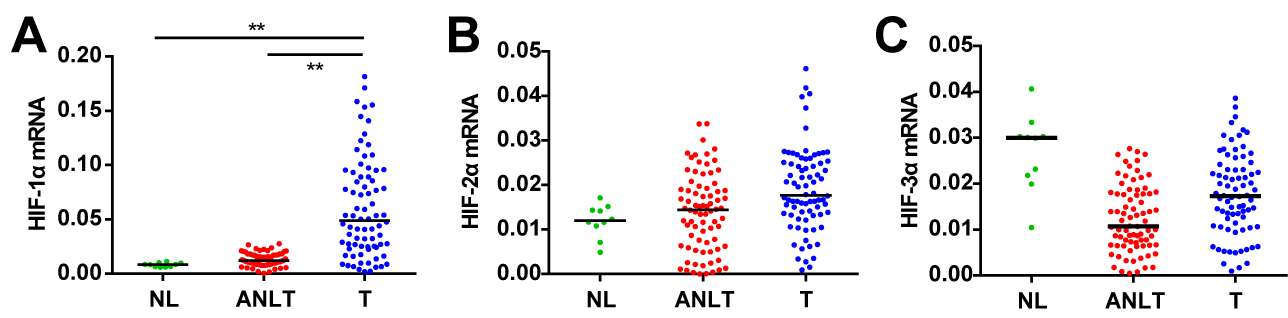

Supplement: Supplementary file 3 — figureS1 [file CTM2-11-e556-s002.tif]

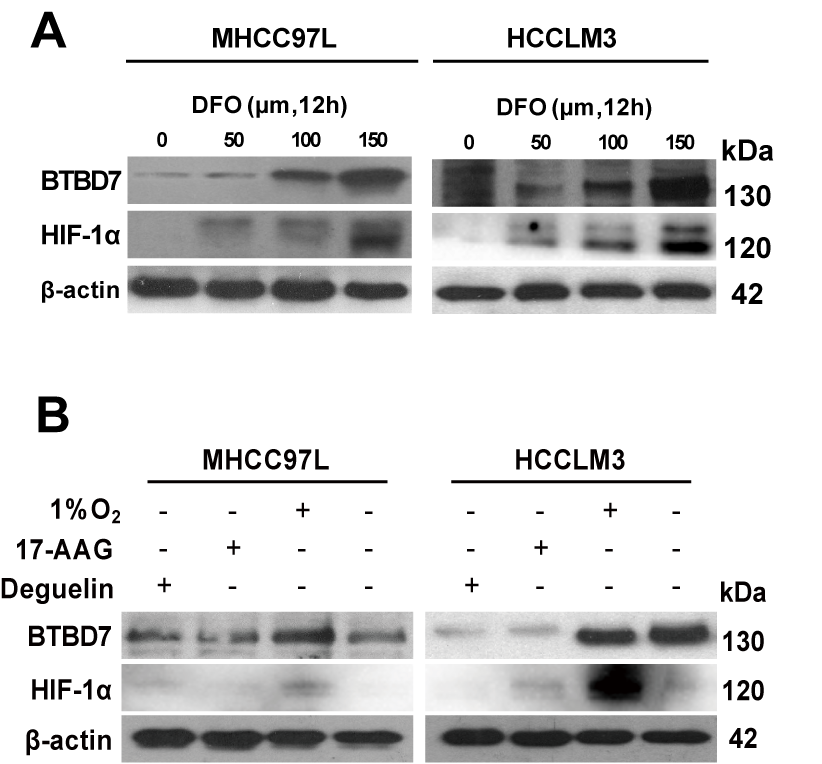

Supplement: Supplementary file 4 — figureS2 [file CTM2-11-e556-s003.tif]

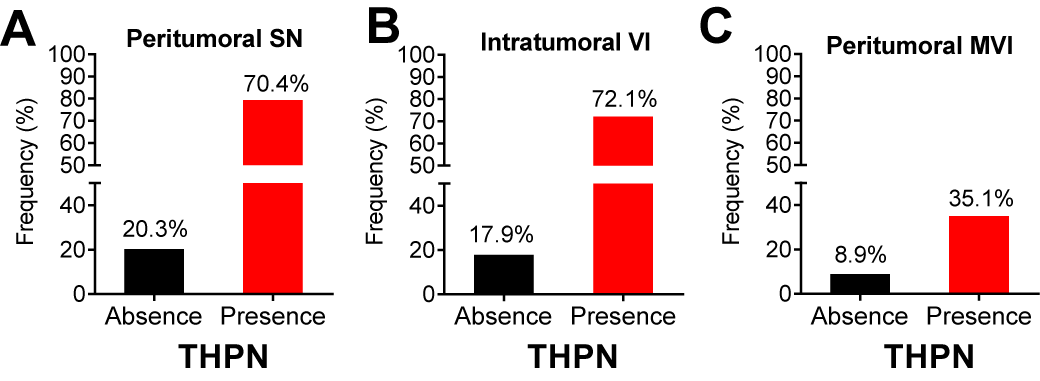

Supplement: Supplementary file 5 — figureS3 [file CTM2-11-e556-s008.tif]

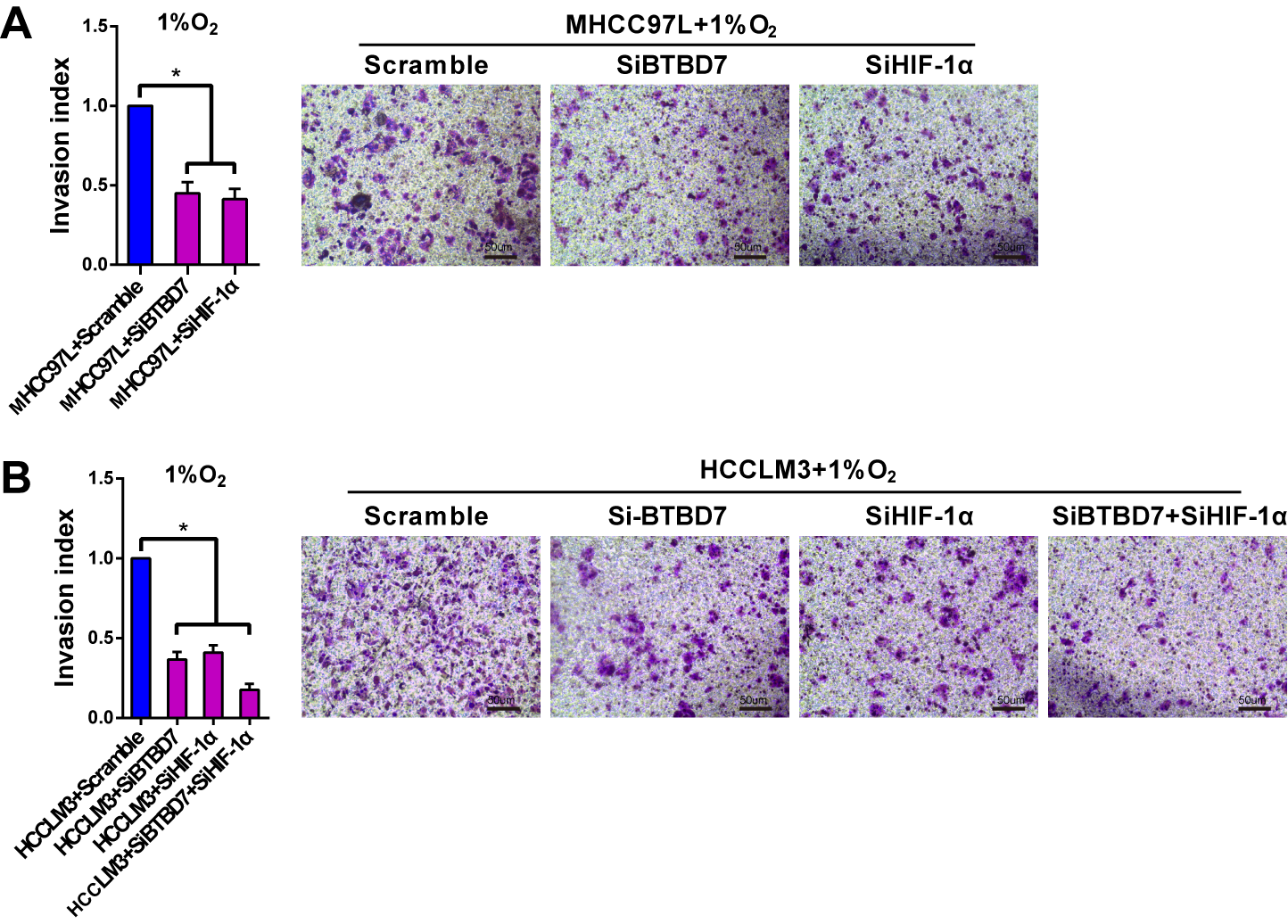

Supplement: Supplementary file 6 — figureS4 [file CTM2-11-e556-s006.tif]

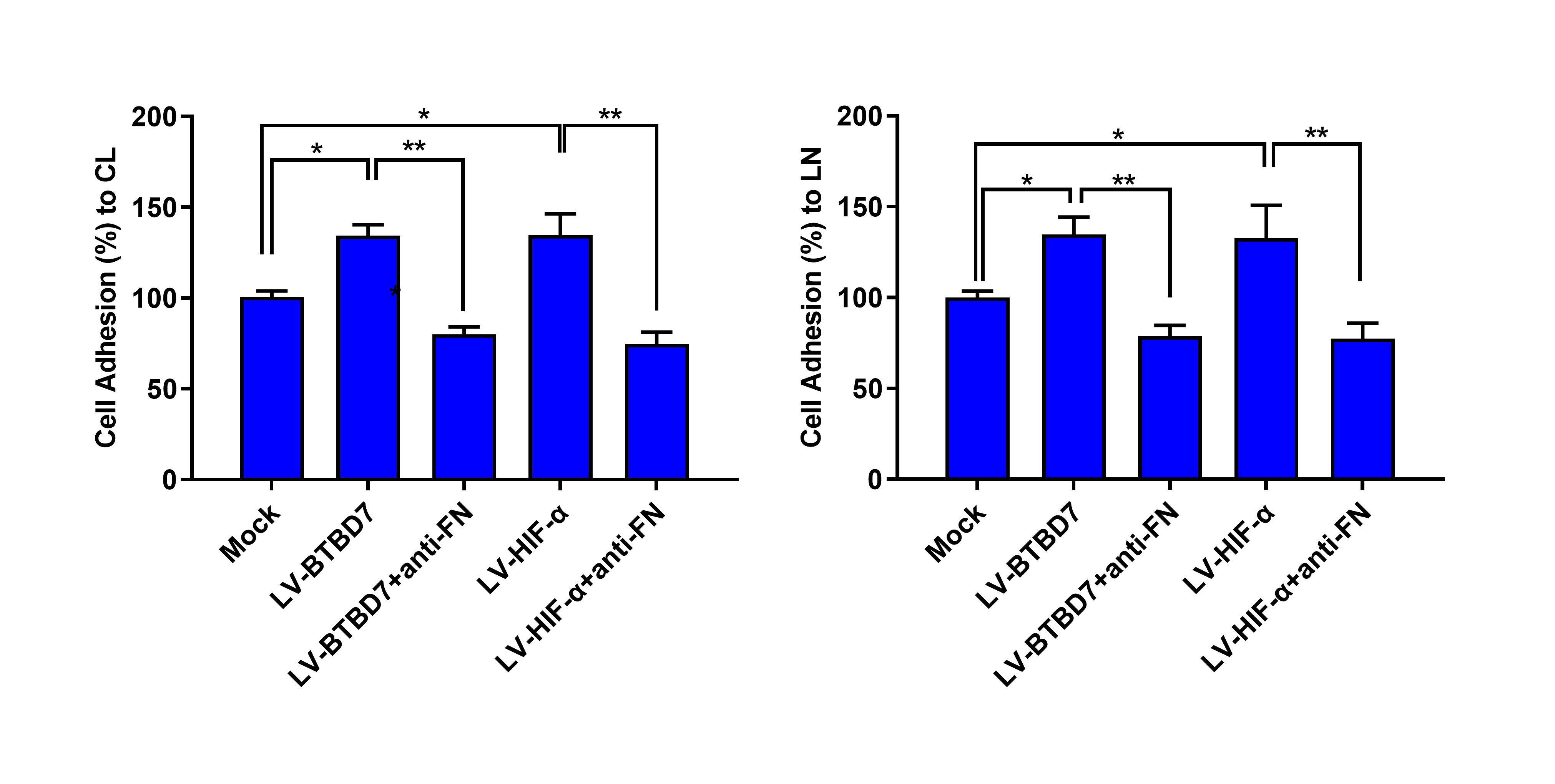

Supplement: Supplementary file 7 — figureS5 [file CTM2-11-e556-s009.tif]

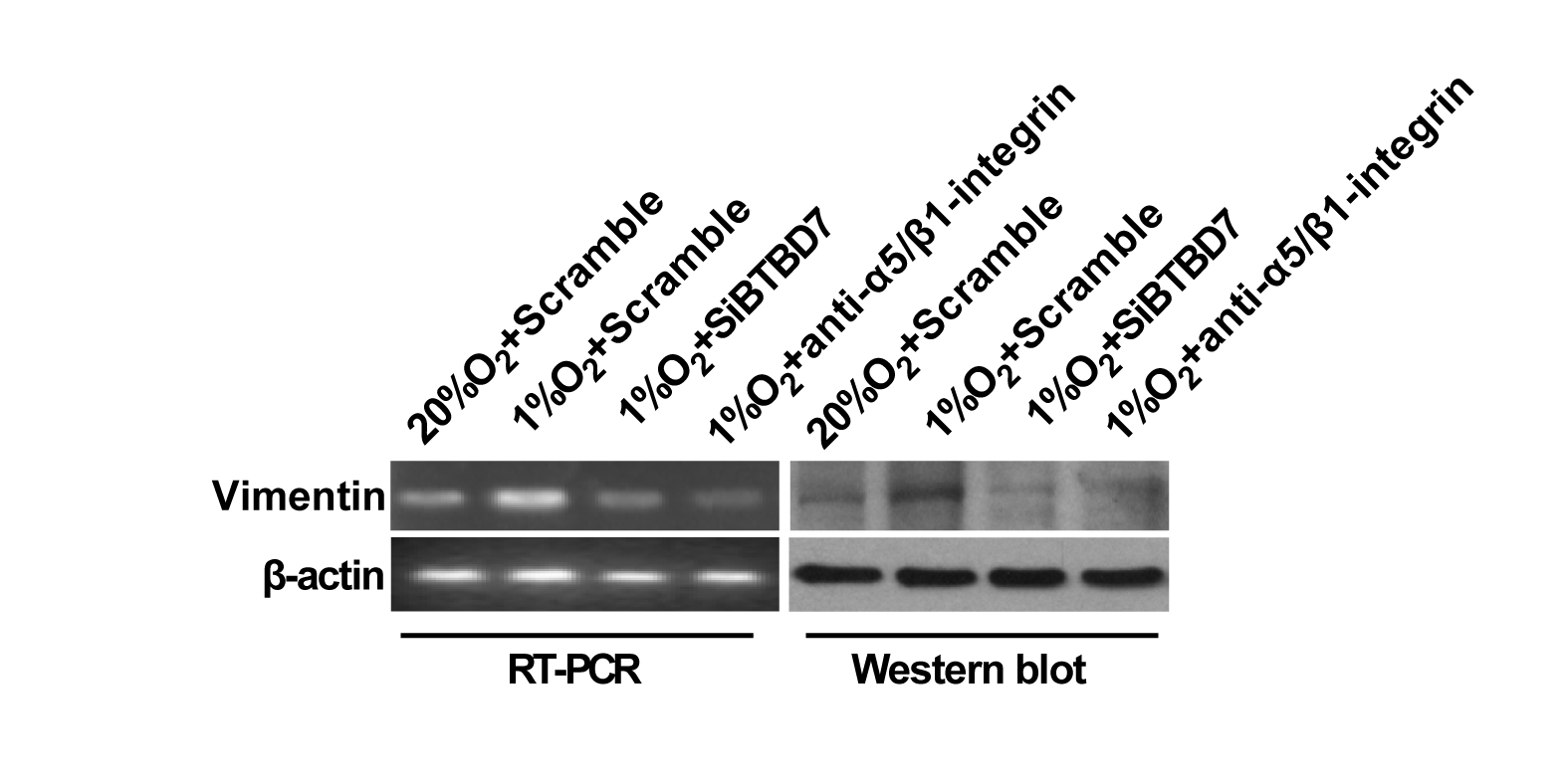

Supplement: Supplementary file 8 — figureS6 [file CTM2-11-e556-s007.tif]

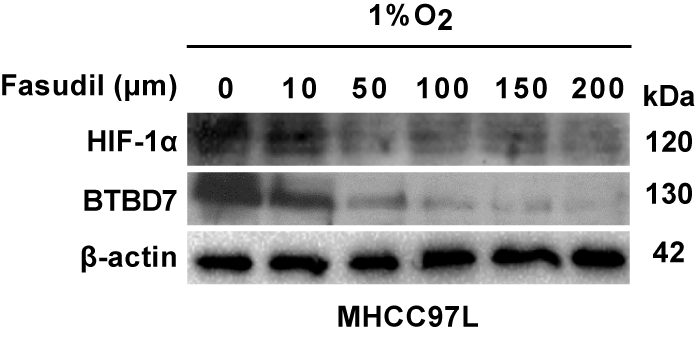

Supplement: Supplementary file 9 — figureS7 [file CTM2-11-e556-s005.tif]
